# RNA-Seq of an LPS-Induced Inflammation Model Reveals Transcriptional Profile Patterns of Inflammatory Processes

**DOI:** 10.3390/life14050558

**Published:** 2024-04-26

**Authors:** Kisung Sheen, Seokho Myung, Dong-Min Lee, Sanghyeon Yu, Yueun Choi, Taeyoon Kim, Jihan Kim, Sang-Gu Ji, Myung-Seo Kim, Wonnam Kim, Yoonsung Lee, Man S. Kim, Yeon-Cheol Park

**Affiliations:** 1 Translational-Transdisciplinary Research Center, Clinical Research Institute, Kyung Hee University College of Medicine, Kyung Hee University Hospital at Gangdong, Seoul 05278, Republic of Korea; kisungsheen@khu.ac.kr (K.S.); tjzh13@khu.ac.kr (S.M.); minya21c@khu.ac.kr (D.-M.L.); sanghyeon99@khu.ac.kr (S.Y.); uag43@khu.ac.kr (Y.C.); taeyuz@khu.ac.kr (T.K.); kknowing@khu.ac.kr (J.K.); jisg8226@khu.ac.kr (S.-G.J.); ylee3699@khu.ac.kr (Y.L.); 2 Department of Biomedical Science and Technology, Graduate School, Kyung Hee University, Seoul 02453, Republic of Korea; 3 Department of Medicine, Kyung Hee University College of Medicine, Seoul 02453, Republic of Korea; 4 Department of Acupuncture & Moxibustion, Kyung Hee University College of Medicine, Kyung Hee University Hospital at Gangdong, Seoul 05278, Republic of Korea; 5 Department of Orthopaedic Surgery, Shoulder & Elbow Clinic, Kyung Hee University School of Medicine, Kyung Hee University Hospital at Gangdong, Seoul 05278, Republic of Korea; myungseo.kim@khu.ac.kr; 6 Division of Pharmacology, School of Korean Medicine, Pusan National University, Yangsan 50612, Republic of Korea; eb75lab@pusan.ac.kr

**Keywords:** LPS-induced inflammation model, whole-blood bulk RNA-seq, core gene list

## Abstract

The LPS-induced inflammation model is widely used for studying inflammatory processes due to its cost-effectiveness, reproducibility, and faithful representation of key hallmarks. While researchers often validate this model using clinical cytokine markers, a comprehensive understanding of gene regulatory mechanisms requires extending investigation beyond these hallmarks. Our study leveraged multiple whole-blood bulk RNA-seq datasets to rigorously compare the transcriptional profiles of the well-established LPS-induced inflammation model with those of several human diseases characterized by systemic inflammation. Beyond conventional inflammation-associated systems, we explored additional systems indirectly associated with inflammatory responses (i.e., ISR, RAAS, and UPR) using a customized core inflammatory gene list. Our cross-condition-validation approach spanned four distinct conditions: systemic lupus erythematosus (SLE) patients, dengue infection, candidemia infection, and staphylococcus aureus exposure. This analysis approach, utilizing the core gene list aimed to assess the model’s suitability for understanding the gene regulatory mechanisms underlying inflammatory processes triggered by diverse factors. Our analysis resulted in elevated expressions of innate immune-associated genes, coinciding with suppressed expressions of adaptive immune-associated genes. Also, upregulation of genes associated with cellular stresses and mitochondrial innate immune responses underscored oxidative stress as a central driver of the corresponding inflammatory processes in both the LPS-induced and other inflammatory contexts.

## 1. Introduction

Inflammation, a multifaceted biological process, involves a diverse array of cells, mediators (such as cytokines and chemokines), and intricate pathways. Irrespective of the underlying cause, inflammation manifests with common symptomatic hallmarks (i.e., redness, fever, and pain) as well as clinical hallmarks, including elevated proinflammatory cytokine levels in serum. However, beyond these well-known features, an abundance of unknown pathways and genes contribute to the orchestration of inflammatory responses [1,2,3]. Among them, some specific pathways and gene networks associated with inflammation may vary depending on the underlying trigger, despite the shared symptomatic and clinical hallmarks.

The lipopolysaccharide (LPS)-induced inflammation model stands as one of the most widely employed systemic models for studying inflammatory-associated processes. Its popularity stems from multiple advantageous features, including cost-effectiveness, reproducibility, and the faithful recapitulation of key inflammatory hallmarks. Researchers have extensively validated the suitability of the LPS model by assessing various proinflammatory markers. Typically, these validations involve monitoring serum levels of critical cytokines such as IL-6, TNF-α, CXCL12, and members of the IL-1 family [4]. In order to comprehensively understand the underlying gene regulatory mechanisms associated with pathogenesis, however, it is imperative to extend validation beyond the clinical hallmarks.

In the intricate landscape of inflammation, peripheral blood turned out to be a central battleground. Immune cells en route to inflamed tissues traverse this crucial conduit, leaving their molecular footprints within the whole-blood transcriptome. Leveraging this rich resource, whole-blood transcriptome analysis provides a cost-efficient avenue to unravel the cues underlying inflammatory-associated mechanisms. Besides, beyond exploring systems, pathways, and gene regulatory networks directly implicated in inflammation-associated functional mechanisms, recent research has reported on alterations of additional factors indirectly involved in inflammatory responses [5]. These factors encompass Integrated Stress Responses (ISR), Renin-Angiotensin-Aldosterone System (RAAS), and Unfolded Protein Responses (UPR).

We harnessed whole-blood bulk RNA-seq data, a cost-effective and clinically relevant resource, and rigorously compared the transcriptional profile patterns of our well-established LPS-induced inflammation model with those of several human diseases characterized by systemic inflammation. Moreover, our study extended beyond the conventional immune-associated systems or pathways through additional exploration of those such as ISR, RAAS, and UPR indirectly associated with inflammatory responses. In this study, we propose that cross-condition validation at the transcriptomic level is essential since only a limited number of studies have investigated whether the LPS-induced inflammation model faithfully recapitulates gene expression profiles at the RNA level. Our primary objective was to assess the model’s suitability for elucidating the molecular mechanisms underlying inflammation triggered by diverse conditions (i.e., SLE patients, dengue infection, candidemia infection, and S. aureus exposure). For this cross-condition validation of the LPS-induced, not only basic analysis approaches such as GO analysis or GSEA, but comparative analysis through alteration patterns of either gene expression or activity level of the gene group using the customized core gene list were applied [5]. This comprehensive approach has the potential to offer a holistic view of LPS-induced inflammation and its diverse applications, thereby illuminating its multifaceted nature.

## 2. Materials and Methods

### 2.1. Animal (Mouse) Preparation

Adult male SD rats (8 weeks old, weighing 200–250 g, from Raonbio, Youngin, Korea) were individually housed with access to water and food ad libitum. The room maintained a 12-h light/dark cycle and remained at a constant temperature of 21–24 °C. The experimental animals underwent a one-week adaptation period before testing. All experiments were conducted following the Guide for the Care and Use of Laboratory Animals [6], and all procedures were approved by the Animal Care and Use Committee of Kyung Hee University Hospital at Gangdong (approval number: KHNMC AP 2022–012).

### 2.2. Lipopolysaccharide (LPS)-Induced Inflammation Model

Lipopolysaccharide (LPS) was procured from Sigma (St. Louis, MO, USA). To establish an acute inflammation model, we intraperitoneally injected 5 mg/kg LPS, following a previously described method with minor adjustments [7]. The study involved two groups, each comprising five animals: (i) Control group: received intraperitoneal injections of phosphate-buffered saline (PBS) at the same dose as the experimental group; (ii) LPS-injected group: received intraperitoneal injections of LPS. Remarkably, no significant systemic side effects resulting from LPS infection, such as changes in body weight or increased mortality, were observed during our experiments. At 6 h post-LPS injection, the rats were anesthetized, and 2.5–3 mL of blood was obtained via cardiac blood sampling for use in the experiment. Blood samples (2.5 mL) were collected using PAXgene Blood RNA Tubes (BRTs) from PreAnalytiX/BD. After 5–10 inversions, the BRTs were refrigerated at 4 °C for up to 5 days to stabilize the RNA.

### 2.3. Library Preparation

The Quant-IT RiboGreen assay was employed to determine the total RNA concentration, with the DV200 value (representing the percentage of RNA fragments > 200 bp) serving as an indicator of RNA sample quality. For library construction, we initially fragmented 100 ng of total RNA into smaller pieces, followed by reverse transcription into first-strand cDNA. Subsequently, second-strand cDNA synthesis occurred, and the resulting products underwent purification and enrichment via PCR to create the cDNA library. To specifically capture human exonic regions, we adhered to the Agilent SureSelect Target Enrichment protocol with the SureSelect XT HS2 RNA Reagent Kit. A total of 250 ng of the cDNA library, combined with hybridization buffers, blocking mixes, RNase block, and 5 µL of the SureSelect all exon capture library, underwent thorough washing and a second round of PCR amplification. The purified product was quantified using KAPA Library Quantification kits designed for Illumina sequencing platforms. Library quality assessment was performed using the TapeStation D1000 ScreenTape, and the indexed libraries were subsequently submitted for paired-end (2 × 100 bp) sequencing on an Illumina NovaSeq (Illumina, Inc., San Diego, CA, USA).

### 2.4. Public Data Preparation

To compare the core gene regulatory patterns of the LPS-induced and inflammation-associated conditions, we downloaded four publicly available RNA-seq datasets from Gene Expression Omnibus (GEO): (i) GSE112087: systemic lupus erythematosus (SLE) patient blood samples (31 SLE samples vs. 29 healthy samples); (ii) GSE140809: acute dengue infection patient blood samples (68 dengue samples vs. 68 convalescent samples); (iii) GSE176260: fungal (candidemia) infection patient blood samples (58 candidemia samples vs. 15 healthy samples); (iv) bacterial (Staphylococcus aureus) infection patient blood samples (4 S. aureus samples vs. 4 healthy samples).

### 2.5. RNA-seq Preprocessing

We obtained gene expression profiles from ten independent libraries; six of them were LPS-induced samples, along with four corresponding control samples. For those ten samples, the paired-end sequencing reads were generated using the Illumina NovaSeq platform. Quality control involved Trimmomatic v0.38, which removed adapter sequences and trimmed low-quality bases. For alignment, we used STAR (v2.7.3a) [8] and HTSeq-count (v0.12.4) [9] to map RNA-seq reads from the ten libraries to the GRCm39 reference genome and its annotation. Gene expression levels were quantified as counts, and normalization using the DESeq2 package [10] with VST (Variance Stabilizing Transformation) was executed. 

### 2.6. Analysis of Inflammatory Regulation

Following the initial preprocessing steps, we performed standard differential analysis and gene ontology (GO) analysis by employing R packages, specifically EnhancedVolcano [11] and clusterProfiler [12]. Also, we explored associations between functional pathways using Gene Set Enrichment Analysis (GSEA) [13]. The results were visualized using Cytoscape [14] in conjunction with Enrichment-Map [15]. In these analysis approaches (i.e., Volcano, GO, and GSEA), we deliberately employed a gene list intricately linked to inflammation. This gene list was meticulously curated by extracting genes associated with the biological process (BP) category of ontology gene sets through the keyword inflammation from the Molecular Signatures Database (MSigDB) [16]. Next, we conducted additional analyses, including the construction of gene expression profile heatmaps and the exploration of inflammatory-specific pathway enrichment. For these latter aspects, we relied on a custom-designed core inflammatory gene list, along with its corresponding inflammatory pathways and modules, as previously described in [5]. Additionally, we implemented Fast Gene Set Enrichment Analysis (fGSEA) [17] with its Nominal Enrichment Score (NES) to enhance our understanding of the inflammatory landscape. These methodological choices were made to ensure robustness and accuracy in our study of inflammation-related mechanisms.

## 3. Results

### 3.1. Inflammation-Associated Genes of the LPS-Induced Model Were Upregulated 

**Figure 1:**
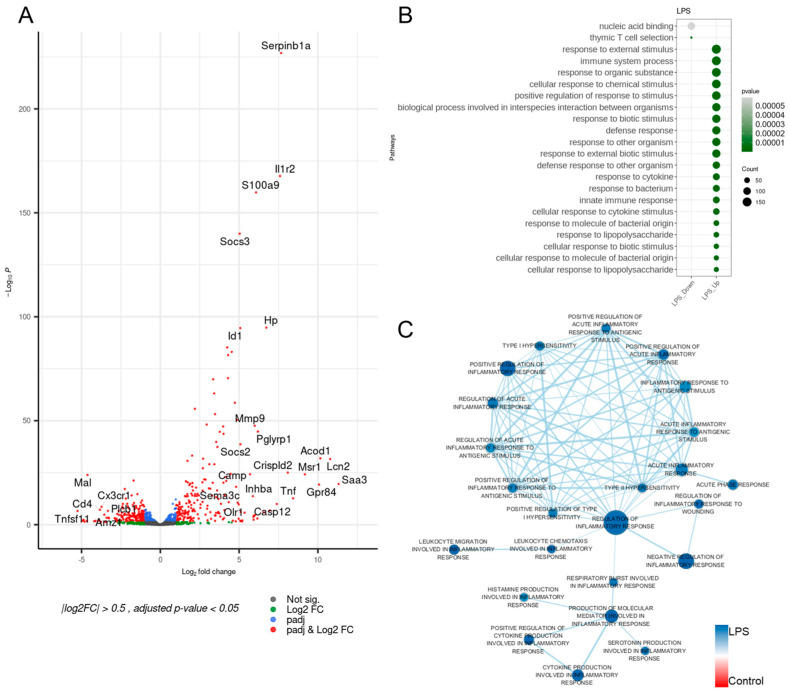
Differentially expressed genes and enriched pathways. (**A**) Differentially expressed genes in the LPS-induced inflammation model compared to the control group (*p*-value < 0.05 and log2 fold-change > 2). (**B**) Inflammation-associated pathways in the LPS-induced inflammation model compared with the control (*p*-value < 0.1 and *q*-value < 0.25). (**C**) Network visualization of enriched pathways from the LPS-induced inflammation model compared with the control. Enriched pathways in the LPS-induced inflammation model are indicated in blue, while pathways enriched in the control are shown in red. Each size of node corresponds to the size of the gene set belonging to the corresponding pathway. Since edges represent the similarity coefficient between connected nodes, thicker lines indicate a higher degree of association.

To explore gene regulatory characteristics associated with inflammatory responses in the LPS-induced model, differentiated from the control, we initially performed standard differential analyses, including GO analysis, utilizing specifically selected inflammation-associated genes acquired from MSigDB. The volcano plot in Figure 1A comparing the LPS-induced and normal (i.e., control) still showed a large amount of differentially expressed genes (DEGs), although the inflammation-associated genes were applied. Also, some turned out to be known genes for typical inflammatory responses: (i) Serpinb1a, acknowledged as a negative regulator of the immune system and documented to be downregulated in tropical pulmonary eosinophilia, was overexpressed in the LPS-induced [18]; (ii) the expression level of Il1r2, widely known for encoding a decoy receptor for interleukin-1, was high in the LPS-induced; (iii) Acod1, supposed to play a role in iron homeostasis and immune modulation of LPS-stimulated macrophage, showed a high expression level in the LPS-induced [19]. While having sufficient DEGs on both up- and down-regulated LPS-induced pathways, most of the differentially enriched functional pathways were displayed on the upregulation side, as depicted in Figure 1B. This indicated that downregulated DEGs from the LPS-induced were probably not functionally associated with each other. Along with some functional pathways associated with immune systems, many different types of stimulus-associated pathways (i.e., response_to_external_stimulus) were strongly and positively enriched. 

To further explore the LPS-induced-specific characteristics associated with inflammatory responses, we implemented GSEA using the same inflammatory-associated gene list from MSigDB. As depicted in Figure 1C, in this analysis, one huge cluster with all nodes in upregulation of the LPS-induced regulation containing regulation_of_inflammatory_response as the central node was detected. Within the cluster, roughly thirteen nodes displayed relatively more considerable connections to each other, where the thirteen nodes were composed of approximately three different types of inflammatory-associated functions: (i) stimulus-associated terms, including responses_to_antigenic_stimulus; (ii) hypersensitivity-associated terms, such as type_I or type_II_ hypersensitivity; (iii) terms implicated in acute_inflammatory_responses.

### 3.2. The LPS-Induced Model Disclosed Considerable Upregulations in Innate Immunity

While lipopolysaccharide (LPS) is widely recognized as a potent inducer of inflammation, there has been a lack of specific investigations into its underlying mechanisms at the gene-by-gene level. Also, although more than 1300 genes are already known to be directly or indirectly associated with inflammatory responses, selective genes among those genes play key roles in inflammatory processes for selective diseases differently.

**Figure 2:**
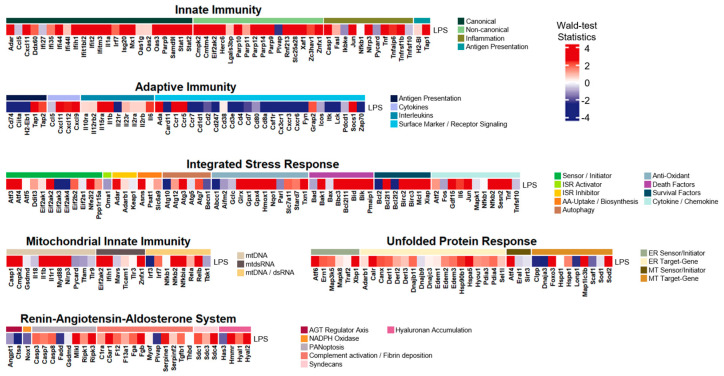
Heatmap of the core inflammatory gene list with Wald-test statistics comparing the LPS-induced versus the control. Transcriptional profile alterations (as Waldtest statistics) of the inflammatory genes selectively organized by Topper and Guarnieri et al. (2023) [5] were visualized. Red indicates upregulation, and blue indicates downregulation in the LPS-induced model.

To better understand the LPS-induced-associated transcriptional profiles implicated in inflammatory responses through specific gene-alteration patterns, we explored differential expression levels of the custom-made core-inflammatory gene list adopted from [5] as depicted in Figure 2. Overall, many inflammatory modules revealed elevated expressions, while only a few modules (i.e., surface marker/receptor signaling in adaptive immunity or NADPH oxidase in RAAS) were suppressed. The most upregulated inflammatory system was innate immunity, unveiling that 69% of the gene list had Wald-test statistics exceeding 2.0. Some inflammatory modules also revealed remarkable elevations (i.e., Wald-test statistics > 2.0); (i) non-canonical in innate immunity as 81%; (ii) cytokines in adaptive immunity as 75%; (iii) death factors in ISR as 75%; and (iv) survival factors in ISR as 71%. adaptive immunity, however, exhibited the most significant downregulation, with 47% of the gene list showing considerable suppression. Specifically, within its module, surface marker/receptor signaling, 62% of the gene list had Wald-test statistics lower than −2.0.

### 3.3. The LPS-Induced Model Exhibited Extraordinary Common Expression Patterns with SLE

**Figure 3:**
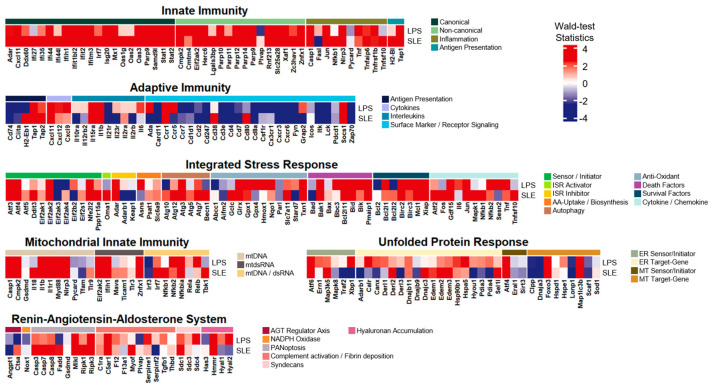
Heatmap of the core inflammatory gene list with Wald-test statistics comparing the LPS-induced model and SLE. Transcriptional profile alterations (as Wald-test statistics) of the inflammatory genes selectively arranged by Topper and Guarnieri et al. (2023) [5] were visualized. Red indicates upregulation, and blue indicates downregulation in either the LPS-induced model or SLE.

Based on the core transcriptional profiles of the LPS-induced acute inflammation model, we compared the gene expression patterns of the core gene list between the LPS-induced and multiple conditions. The first case chosen to be compared was systemic lupus erythematosus (SLE), which is a representative autoimmune disease characterized by systemic inflammation (Figure 3). The RNA-seq data downloaded for this comparison contained whole-blood transcription samples of both 31 SLE patients and 29 healthy donors (GSE112087). On the whole, this comparison with SLE patients revealed that gene alteration patterns were sufficiently indistinguishable across the two different datasets. From the comparison, several genes with relatively substantial elevations in common for both groups (i.e., Wald-test statistics > 6.0) were shown: (i) innate immunity: Ifih1, Ifit2, Oas3, Parp9, Stat1, Stat2, Herc6, Parp14, Parp9, Znfx1, Casp1, and Tap1; (ii) adaptive immunity: Tap1 and Ccr1; (iii) mitochondrial innate immunity: Casp1, Myd88, Ifih1, and Znfx1; (iv) RAAS: Mlkl; (v) ISR: Nfe2l2 and Glrx. Among the gene list, some were reported in previous studies as follows: (i) Ifih1 was related to viral resistance in children [20]; (ii) Ifit2 was involved in resistance to viral infection such as influenza virus [21]; (iii) Znfx1 was associated with susceptibility to viral infections [22]; (iv) transcription of Tap1 could be rapidly upregulated in response to pro-inflammatory cytokines such as type-I IFN, IFN-γ, and TNF-α [23]; (v) Ccr1 could contribute to viral infection by activating inflammation in infection situations [24]; (vi) Myd88 was associated with IL-1 signaling [25].

The most similar inflammatory system turned out to be innate immunity. 67% of the core gene list in innate immunity were upregulated (i.e., Wald-test statistics > 2.0) in both the LPS-induced and the SLE patient samples, where canonical and non-canonical presented 71% and 75% common upregulations, respectively. Other inflammatory modules also displayed relatively more significant upregulations in common for both groups: (i) survival factors in ISR as 57%; (ii) mtDNA in mitochondrial innate immunity as 55%. Also, other inflammatory modules divulged relatively more common patterns, whether commonly upregulated or downregulated (i.e., either Wald-test statistics > 2.0 or Wald-test statistics < 2.0), in both groups: (i) surface marker/receptor signaling in adaptive immunity as 54%; (ii) antigen presentation in adaptive immunity as 80%; (iii) MT target gene in UPR as 56%. However, we observed unmatched patterns of expression alterations across the gene list. Among the inflammatory modules having these incomparable patterns, interleukins in adaptive immunity and hyaluronan accumulation in RAAS demonstrated 56% and 75% differences in alteration (i.e., Wald-test statistics > 0 and Wald-test statistics < 0). These two cases were the only modules with higher percentages of unmatched alterations exceeding 50%.

### 3.4. The LPS-Induced Model Revealed Exceptional Common Expression Patterns with Dengue Virus Infection

**Figure 4:**
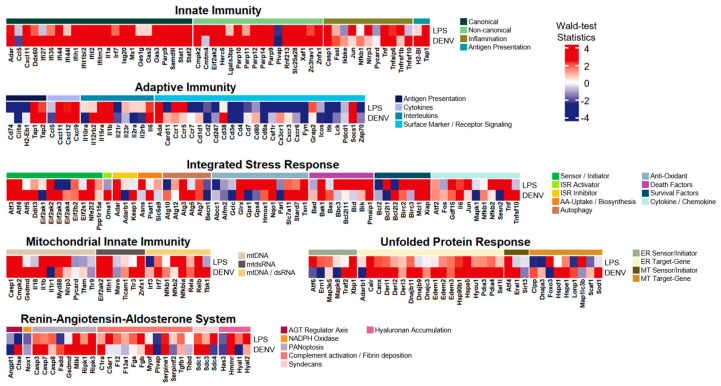
Heatmap of the core inflammatory gene list with Wald-test statistics comparing the LPS-induced inflammation model and the dengue virus infection. Transcriptional profile alterations (as Wald-test statistics) of the inflammatory genes selectively arranged by Topper and Guarnieri et al. (2023) [5] were visualized. Red indicates upregulation, and blue indicates downregulation in either the LPS-induced model or dengue virus infection.

In line with the comparison to the SLE patient samples, we selected dengue virus infection blood samples to examine transcriptional profile patterns intimately associated with inflammatory responses (Figure 4). The dengue infection samples downloaded from GSE140809 consisted of whole blood samples composed of 68 pediatric patients and 68 convalescent (i.e., 14–22 days post-infection) controls. In the comparison, multiple genes with relatively considerable elevations in common for both groups (i.e., Wald-test statistics > 6.0), where many of these were overlapping with those from the comparison with the SLE, were demonstrated: (i) innate immunity: Ifih1, Ifit2, Ifitm3, Oas3, Parp9, Stat1, Stat2, Herc6, Parp14, Parp9, Znfx1, Casp1, and Tap1; (ii) adaptive immunity: Tap1 and Socs1; and (iii) mitochondrial innate immunity: Casp1, Ifih1, and Znfx1, (iv) RAAS: Mlkl, (v) ISR: Glrx and Txn1.

While exhibiting overlapping up- or down-regulation patterns in general, innate immunity was uncovered to be the second most identical inflammatory system, where 62% of the core gene list were considerably upregulated (i.e., Wald-test statistics > 2.0) in both the LPS-induced and the dengue infection samples. Two inflammatory modules, non-canonical in innate immunity and cytokines in adaptive immunity, disclosed exceptional resemblance by sharing 75% of common upregulations between both the LPS-induced and the dengue infection. Additionally, two other inflammatory modules revealed significant common patterns, including upregulation and downregulation (i.e., either Wald-test statistics > 2.0 or Wald-test statistics < 2.0), within non-canonical in innate immunity and antigen presentation in adaptive immunity by 81% and 60%, respectively. However, this comparison also presented unparalleled alteration patterns over the gene list in some inflammatory modules, as follows: (i) ER sensor/initiator in UPR by 50%; (ii) MT target gene in UPR by 67%; and (iii) AGT regulator axis in RAAS by 50%.

### 3.5. The LPS-Induced Model Disclosed Significant Common Expression Patterns with Candidemia Infection

**Figure 5:**
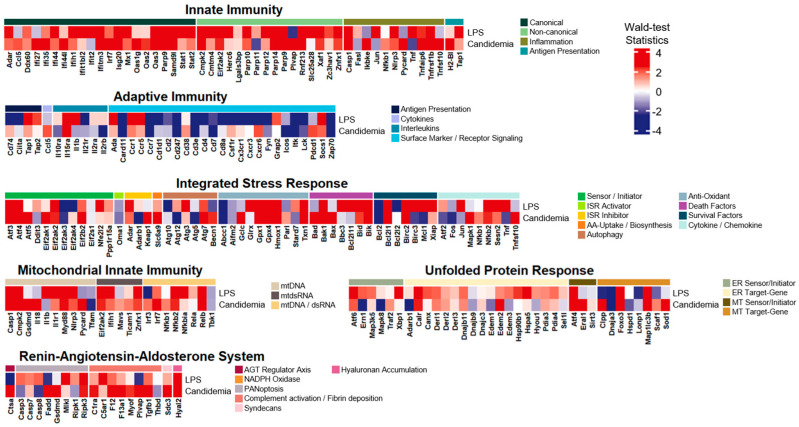
Heatmap of the core inflammatory gene list with Wald-test statistics comparing the LPS-induced inflammation model and Candidemia. Transcriptional profile alterations (as Wald-test statistics) of the inflammatory genes selectively arranged by Topper and Guarnieri et al. (2023) [5] were visualized. Red indicates upregulation, and blue indicates downregulation in either the LPS-induced model or Candidemia.

Along with the SLE patient samples and the dengue infection samples, we also included fungal (candidemia) infection samples to scan their differential expression patterns with the LPS-induced (Figure 5). The candidemia infection samples were downloaded from GSE176260, which is RNA-seq data on peripheral blood from 58 hospitalized patients with candidemia infection and 15 controls. According to this comparison, a few genes (Ifitm3 in innate immunity, Relb in mitochondrial innate immunity, and GPX4 in ISR) with relatively considerable upregulations in common for both groups (i.e., Wald-test statistics > 5.0) as stated in previous studies are as follows; (i) Ifitm3 in innate immunity was intimately associated with antiviral protection [26]; (ii) GPX4 in ISR may suggest cellular protection in response to oxidative stress [27].

This comparison between the LPS-induced and the candidemia infection samples also unveiled indistinguishable alteration patterns across all the gene lists of six inflammatory systems, as the SLE patient samples and the dengue infection reported. While some inflammatory modules indicated relatively significant comparable patterns between the two different groups (i.e., anti-oxidant in ISR with statistics, either Wald-test statistics > 2.0 or Wald-test statistics < 2.0), this comparison generally revealed modest likeness across the gene list between the two groups. Despite the insignificant comparability with variations between the two groups, commonalities in alteration patterns between the groups were still maintained with cut-offs of either Wald-test statistics > 0 or Wald-test statistics < 0. Based on this cut-off, the similarity percentage of commonly upregulated or downregulated genes over all the gene lists between the LPS-induced and the candidemia infection samples turned out to be 59%, while the other comparisons with either SLE samples or dengue virus samples were 62% or 63%, respectively.

### 3.6. The LPS-Induced Model Divulged Considerable Common Expression Patterns with Exposure to Heat Killed S. aureus

**Figure 6:**
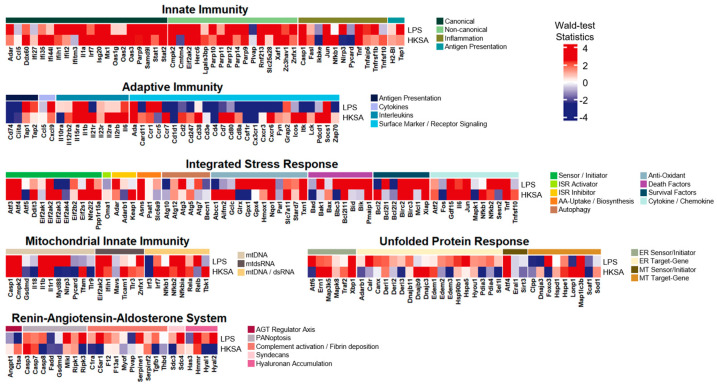
Heatmap of the core inflammatory gene list with Waldtest statistics comparing the LPS-induced model and exposure to the heat-killed S. aureus. Transcriptional profile alterations (as Wald-test statistics) of the inflammatory genes selectively arranged by Topper and Guarnieri et al. (2023) [5] were visualized. Red indicates upregulation, and blue indicates downregulation in either the LPS-induced model or exposure to HKSA.

In addition to the SLE, the dengue infection, and the candidemia infection cases, we additionally took into account bacterial (*Staphylococcus aureus*) exposure samples to examine gene expression alteration patterns for comparison with those of the LPS-induced (Figure 6). A publicly available RNA-seq dataset (GSE237960), four samples of human whole blood exposure to heat-killed *S. aureus* (HKSA), along with four controls, was downloaded to be analyzed. In this comparison between the LPS-induced and the S. aureus exposure, some genes were significantly and commonly upregulated for both groups (i.e., Wald-test statistics > 6.0); (i) innate immunity: Herc6 and Casp1; (ii) adaptive immunity: Il1b and Socs1; (iii) mitochondrial innate immunity: Casp1 and Il1b; (iv) ISR: Nqo1 and Txn1. From this list, Socs1, the suppressor of cytokine signaling 1, was described as being negatively correlated with the magnitude of inflammation. A recent study conducted blood transcriptome profiling from the peripheral blood of atopic dermatitis patients and classified them into two endotypes—eosinophil high/low. Of these two endotypes, Socs1 was upregulated in the former endotypes [28].

Similar alteration patterns across all the inflammatory systems were also disclosed, consistent with the aforementioned fungal infection case. Some inflammatory modules, such as non-canonical in innate immunity, antigen presentation in adaptive immunity, and ER sensor/initiator in UPR, revealed significant comparable patterns between the two groups (Wald-test statistics > 2.0 or Wald-test statistics < 2.0). While this comparison demonstrated moderate resemblance across the two groups alike in the dengue infection, the similarity percentage of commonly upregulated or downregulated genes over all the gene lists between the LPS-induced and the S. aureus exposure was computed at 64% (Wald-test statistics > 0 or Wald-test statistics < 0).

### 3.7. Innate Immunity and Mitochondrial Innate Immunity Demonstrated the Most Similar Activation Patterns across Four Distinct Inflammatory Conditions

In attempts to compare alteration patterns of inflammatory activation levels between the LPS-induced and the other four different RNA-seq datasets (the SLE patient, the dengue infection, the candidemia infection, and the S. aureus exposure), we estimated the activation levels of all the inflammatory modules by evaluating their corresponding normalized enrichment scores (NES) using fGSEA. Based on the NES values as estimated collective dynamics of specific inflammatory-associated gene-groups, we compared all five different conditions across all thirty inflammatory modules by examining the values of the modules to determine whether they were comparable or different over the different conditions.

**Figure 7:**
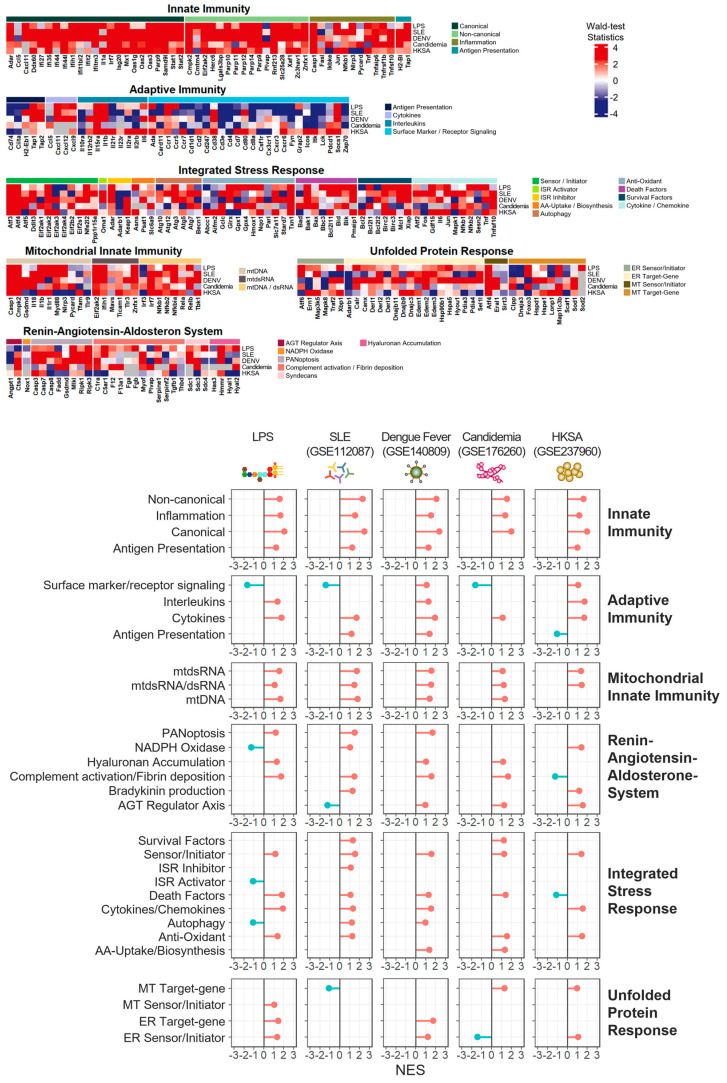
Combined heatmaps with Wald-test statistics and lollipop plots with statistically significant changes by normalized enrichment score (NES) for the LPS-induced inflammation model, SLE (GSE112087), DENV (GSE140809), Candidemia (GSE176269), and exposure to HKSA (GSE237960).

According to the patterns shown in Figure 7, innate immunity-associated systems, including innate immunity and mitochondrial innate immunity revealed the most remarkable resemblance across all the five different conditions. Specifically, inflammatory modules such as canonical and non-canonical in innate immunity demonstrated commonly upregulated activation levels over the five groups. Modules such as mtdsRNA and mtdsRNA/dsRNA in mitochondrial innate immunity also indicated common upregulated patterns across the five distinct conditions. While displaying less comparability across the five groups than innate immunity-associated systems, adaptive immunity still exhibited marked likeness, particularly on its module, cytokines. Surface marker/receptor signaling in adaptive immunity also revealed similarities across four of the groups, except for dengue infection. 

However, the other inflammatory systems (i.e., RAAS, ISR, and UPR) did not introduce agreement across the five different conditions in general, although considerable alikeness of some inflammatory modules was detected: (i) RAAS: complement activation/fibrin deposition and (ii) ISR: sensor/initiator, death factors, cytokines/chemokines, and anti-oxidant. Among all six inflammatory systems, UPR turned out to be the most disparate system across all five conditions.

## 4. Discussion

We have investigated the inflammatory-associated gene regulatory patterns of the LPS-induced acute inflammation mouse model to study its underlying inflammatory-associated functional processes and determine whether this inflammation model can be used to study the inflammatory processes of various biomedical conditions, including diseases, infections, or exposures. To compare specific gene expression alterations of the LPS-induced with those of several other conditions, we downloaded representative RNA-seq datasets of four different conditions, such as the SLE patient, the dengue infection, the candidemia infection, and the S. aureus exposure. In this comparison analysis, we primarily applied the custom-made core inflammation gene list to track their expression alteration patterns and also the gene set enrichment analysis with the gene list across all the different conditions.

Our initial analyses (i.e., differential analysis, GO analysis, and GSEA) were conducted on the LPS-induced inflammatory mouse model and revealed many different alterations mainly associated with innate immunity and adaptive immunity. While these analyses still showed diverse links between known or typical functional pathways and gene regulatory alterations, they neither provided connections to previously undiscovered pathways nor introduced specific gene lists for a better interpretation of the underlying mechanisms associated with inflammatory processes (Figure 1). Therefore, based on the findings from Figure 2, our primary analysis approach focused on exploring gene expression alteration patterns as well as activation level alterations of inflammatory modules using the adopted custom-made core gene list. We initially applied this approach to the LPS-induced and subsequently extended it to contain four other distinct inflammatory conditions: systemic lupus erythematosus (SLE), dengue virus infection, candidemia (fungal infection), and heat-killed S. aureus (bacterial infection).

The most noteworthy findings from our investigation are gene lists that displayed relatively significant common up- or down-regulation alterations among known genes associated with each inflammation-associated condition (i.e., Wald-test statistics > 2.0 for both cases). The LPS-induced model expressed known innate immune marker genes in systemic auto-immune disease, systemic lupus erythematosus. Cxcl11, Ifih1, Isg20, Oas3, Parp9, Stat1, Stat2, Herc6, and Casp1 are associated with type 1 interferon signaling pathways and mononuclear cell infiltration [29,30,31,32,33,34,35,36]. Expressions of Tap1, Tap2, and Socs1 implicated in cytokines and interleukin were commonly increased [23,28] and another common elevation of Adar was found to be involved in RNA editing in SLE [37]. However, some known adaptive immune marker genes associated with leukocyte migration in SLE, such as Cd74 and Cxcr6, were downregulated in common [38,39]. Others, such as Ccr7, Cd4, Cxcr6, Fyn, Lck, and Zap70, were also known to decrease in SLE, as observed [39,40,41,42,43,44]. 

The LPS-induced model also shared similar marker genes with the systemic viral infection, dengue infection. Gene expressions associated with antiviral activity, including Znfx1, Adar, Ifi44, Ifih1, Ifit2, Ifitm3, and Isg20 [20,21,22,26,45,46,47,48] were increased in common. Additionally, expressions of Myd88, which is involved in interleukin expression [25,49], and Irf7, implicated in IFN expression [50], were both increased.

In addition, the LPS-induced model exhibited shared marker genes with systemic fungal infection and Candidemia infection. Genes with commonly elevated expressions encompass Ripk3, Parp, and Stat, which are known to be associated with the IFN, MAPK, and NF-kappa-b pathways [51,52,53]. Other genes involved in stress response and cytokine expression were Atf4, Atf6, and Eif2ak2 in common upregulation [54,55,56,57].

Lastly, the LPS-induced model displayed overlapped marker genes with heat-treated S. aureus samples. Their innate immune markers, such as Oas3, Parp9, and Samd9l, are known to be increased in TB infection [58,59,60]. While Eif2ak2 and Herc6 are implicated in bacterial sepsis [61], Rnf213 is associated with ubiquitylation of LPS [62], and Casp1 is a key factor in the inflammatory response [63] in bacterial infection as detected in common upregulation. However, Cd74, an adaptive immune marker, is known to be decreased in Pseudomonas infection [64].

Increased gene expression associated with cellular stresses, such as RAAS, ISR, and UPR, along with increased mitochondrial innate immunity, suggests that those stresses play a key role in necessitating the inflammatory response in the LPS-induced model. Previous studies have shown that LPS can induce hypoxia, which in turn affects macrophages and the autophagy of dendritic cells [65,66]. The increased gene expression of Foxo3, Sod2, Gpx1, and Gpx4 in the LPS-induced model can be associated with the response to oxidative stress [27,67,68]. This pattern of inflammatory gene expression has also been observed in several systemic inflammatory diseases, including SLE, dengue infection, Candidemia infection, and exposure to S. aureus.

The considerable upregulation of both canonical and non-canonical innate immunity across all the modules may indicate its association with initial phases of immune responses that counter invading pathogens, particularly those mediated by reactive oxygen species (ROS) [69]. The upregulation of inflammatory modules such as mtDNA, mtdsRNA, and mtDNA/dsRNA in mitochondrial innate immunity may insinuate the involvement of innate immune responses in cellular damage and stress [70]. The promoted levels of some modules (i.e., PANoptosis, complement activation/fibrin deposition, syndecans, and hyaluronan accumulation) in Renin-Angiotensin-Aldosterone System (RAAS) may be implicated in inflammatory processes associated with cell death [71]. The elevated levels of modules such as ER sensor/initiator and ER target gene in Unfolded Protein Response (UPR) may imply a possible impact on innate immune signaling, immune cell functions, and dealing with oxidative stress in the endoplasmic reticulum [72]. For instance, FOXO3, a member of the FOXO (Forkhead Box O) protein family, promotes expressions of intracellular antioxidant genes, reduces concentrations of various oxidative substances, and induces autophagy to remove damaged cellular components and oxidative agents, thereby possibly enhancing its activity in immediate response to transient oxidative stress [67]. The uplifted levels of some modules (i.e., anti-oxidant, death factors, survival factors, and cytokine/chemokine) in Integrated Stress Response (ISR) may be associated with a wide range of cellular stresses, including oxidative stress. For example, GPX enzymes (i.e., GPX1 or GPX4) [27] utilize glutathione as a cofactor to reduce peroxides within the cell while converting peroxides into less harmful substances. This process implies a cellular protective response to oxidative stress.

One significant limitation of this study may stem from the data utilized for all analyses. As all five datasets analyzed in this study were based on RNA sequencing, our observations were limited to alterations only at the transcriptional level. The interpretations of inflammatory-associated changes across different cases may be less sensitive.

## 5. Conclusions

In conclusion, the inflammatory pathway observed in the LPS-induced inflammatory model aligns with the inflammatory-process patterns traced in other systemic inflammatory diseases. The LPS-induced model exhibits similar inflammatory-process profiles to those observed in patients with SLE, dengue infection, Candidemia infection, and exposure to S. aureus. Specifically, the LPS-induced model expresses known marker genes consistent with the inflammatory-process patterns detected in the comparison disease set, supporting the notion of similarity. Among the samples, an increase in innate immune-related genes, particularly in chemokine expression, was disclosed. Adaptive immune genes implicated in surface presentation, however, demonstrated decreased expression. Furthermore, the upregulation of genes involved in oxidative stress and mitochondrial innate immunity underscored oxidative stress as a primary inducer of inflammation in both LPS-induced conditions and other diseases.

## Data Availability

The data presented in this study are available on request from the corresponding author.
